# Novel Membranes Based on Hydroxyethyl Cellulose/Sodium Alginate for Pervaporation Dehydration of Isopropanol

**DOI:** 10.3390/polym13050674

**Published:** 2021-02-24

**Authors:** Mariia Dmitrenko, Andrey Zolotarev, Vladislav Liamin, Anna Kuzminova, Anton Mazur, Konstantin Semenov, Sergey Ermakov, Anastasia Penkova

**Affiliations:** Department of Analytical Chemistry, Institute of Chemistry, St. Petersburg State University, 7/9 Universitetskaya Nab., 199034 St. Petersburg, Russia; andrey.zolotarev@spbu.ru (A.Z.); lyamin.vlad.322@gmail.com (V.L.); ai.kuzminova@mail.ru (A.K.); a.mazur@spbu.ru (A.M.); semenov1986@yandex.ru (K.S.); s.ermakov@spbu.ru (S.E.); a.penkova@spbu.ru (A.P.)

**Keywords:** sodium alginate, hydroxyethyl cellulose, fullerenol, layer-by-layer assembly, pervaporation dehydration

## Abstract

Membrane methods, especially pervaporation, are quickly growing up. In line with that, effective membrane materials based on biopolymers are required for the industrially significant mixtures separation. To essentially improve membrane transport characteristics, the application of the surface or/and bulk modifications can be carried out. In the present study, novel dense and supported membranes based on hydroxyethyl cellulose (HEC)/sodium alginate (SA) were developed for pervaporation dehydration of isopropanol using several approaches: (1) the selection of the optimal ratio of polymers, (2) the introduction of fullerenol in blend polymer matrix, (3) the selection of the optimal cross-linking agent for the membranes, (4) the application of layer-by-layer deposition of polyelectrolytes on supported membrane surface (poly(sodium 4-styrenesulfonate) (PSS)/poly(allylamine hydrochloride) (PAH) and PSS/SA). Structural and physicochemical characteristics of the membranes were analyzed by different methods. A cross-linked supported membrane based on HEC/SA/fullerenol (5%) composite possessed the following transport characteristics in pervaporation dehydration of isopropanol (12–50 wt.% water): 0.42–1.72 kg/(m^2^h) permeation flux, and 77.8–99.99 wt.% water content in the permeate. The surface modification of this membrane with 5 bilayers of PSS/PAH and PSS/SA resulted in the increase of permeation flux up to 0.47–3.0 and 0.46–1.9 kg/(m^2^h), respectively, with lower selectivity.

## 1. Introduction

From the point of view of environmental impact, industry is one of the most dangerous human activities. In this regard, the control conditions for the industrial development becomes ever more stringent, and the industry is constantly improving its production methods by the use of novel technologies to improve the efficiency and productivity of processes, and make them economically beneficial and environmentally friendly. One of the most advanced and rapidly developing separation technologies are membrane processes, which are also referred to as sustainable processes due to their characteristics: low energy consumption, high selectivity of processes, environmental friendliness, compact modular equipment, and easy automation. Pervaporation, a membrane process capable of the separation of liquid mixtures containing low molecular weight substances, gains steadily growing attention. Pervaporation is a relatively new and advanced separation technology that can be an alternative to traditional separation methods, since it can be used for the separation of azeotropic and isomers mixtures, and close-boiling and thermally unstable substances. Pervaporation is energy effective and does not require the use of an additional toxic intermediate agent for separation, which makes it a cost effective and environmentally friendly process. Pervaporation is actively applied in various sectors of the economy such as medicine, water treatment, energy, chemical, and food industries [1]. In particular, a very important direction for application of pervaporation is the dehydration of alcohols [2,3].

The wide spread system in membrane pervaporation is isopropanol-water mixture contained azeotrope (water–isopropanol 12/88 wt.%) [4] for the sake of alcohol dehydration. At present, due to its good degreasing and cleaning properties and low toxicity, high purity isopropanol is in great demand because it is actively used as a substitute for ethyl alcohol, in cosmetics including perfumery, household chemicals, automotive fluids, glass cleaners, office equipment and electronics, and medicine [5,6,7,8,9]. It is also worth noting that isopropanol is a wide spread solvent and feedstock for the synthesis of important organic substances (such as acetone, hydrogen peroxide, etc.) [10]. It is actively used to clean the accumulated water in a car’s gas tank, which can block the fuel system, since water does not mix with petrochemicals. However, traditional separation methods for isopropanol purification from water, such as distillation, may provide the yield of only 88% isopropanol [11]. For further rectification of alcohol, the use of additional toxic separating agents are required to break the azeotropic mixture, which also further complicates the isopropanol purification and creates an additional separation step. However, pervaporation can easily solve this problem for the dehydration of alcohols without the use of toxic additives [12]. It makes it possible to obtain high-purity isopropanol by correct choice of the membrane.

Nowadays, different types of membranes for alcohol dehydration are developed using various materials. Membranes based on hydrophilic polymers are the most widely applied, due to their universality, simple manufacturing, low costs, high reproducibility of the separation, and high flexibility. There is a possibility to modulate the properties of the membranes using volume and surface modifications. The most commonly used membrane polymer materials for pervaporation dehydration are polyvinyl alcohol [13,14], polyamides and polyimides [15,16,17,18,19], polysulfone [20,21,22], polyacrylonitrile [23,24], chitosan [25,26,27], and aromatic polymers (e.g., polybenzoxazole and polybenzimidazole) [28,29], etc. These membranes are often modified by blending with other polymers [30,31], modifying with various reagents [32], and cross-linking by different methods [2,33,34].

Owing to the existing limitations on the environmental impact of industries and their constant tightening, it is necessary and most relevant to use polymers based on biological materials for the membrane production due to their environmental friendliness, high productivity, and sustainability [35]. This work addresses the development of novel high-performance membranes based on sodium alginate (SA) and hydroxyethyl cellulose (HEC) blend for pervaporation dehydration of isopropanol. SA is a natural polysaccharide extracted from seaweed, which is actively applied in medical [36,37], paper, cosmetic, and food industries [38], and as a membrane material in various membrane processes [39,40,41,42,43,44,45], especially, in pervaporation for dehydration of organic solvents [46,47,48,49,50]. Hydroxyethyl cellulose (HEC) is a carbohydrate polymer, namely, water-soluble cellulose ether with high compatibility with various water-soluble polymers [51,52,53]. This polymer is actively used as a thickener in paints and finishes [51], polymer matrix for the membrane development for the pervaporation catalytic membrane reactor [54,55,56]. However, there are only a few articles concerning pervaporation application of HEC [6,51,53,57,58], and three articles devoted to the application of HEC/SA membranes [6,51,57]. In the work [57], the solution and film blend compatibility of SA and HEC was studied. The dense HEC/SA membranes with 35–40 µm thickness were prepared in various weight ratios of 5/95, 10/90, 20/80, 30/70, 40/60, 60/40, 70/30, and 80/20 with the sequential two-stage cross-linking with glutaraldehyde and urea–formaldehyde–sulfuric acid mixture. The cross-linked SA and blend HEC/SA (10/90) membranes with optimal properties were tested in the pervaporation separation of water/1,4-dioxane and water/tetrahydrofuran (THF) mixtures. The blend membrane demonstrated better transport properties for THF dehydration (10–20 wt.% water) at 30 °C (0.183–0.258 kg/(m^2^h) permeation flux, 99.41–98.59 wt.% water in the permeate) compared with cross-linked SA membrane (0.178–0.217 kg/(m^2^h) permeation flux, 97.12–94.29 wt.% water in the permeate). These authors also attempted to improve performance of blend HEC/SA membranes by modification with ZSM-5(40) zeolite for pervaporation dehydration of isopropanol [6]. Blend dense HEC/SA membranes (35–40 µm thickness) were prepared with the following ratios 5/95, 10/90, and 20/80 wt.% with the use of the same two-stage cross-linking method. Based on pervaporation data, the HEC/SA (10/90) membrane was chosen as the best for further modification with 10 wt.% ZSM-5(40) zeolite, which resulted in increased membrane flux without affecting selectivity. The modified HEC/SA(10/90)/ZSM-5(40) (10%) membrane had 0.2–0.25 kg/(m^2^h) permeation flux and 99.97–99.63 wt.% water in the permeate in pervaporation dehydration of isopropanol (10–15 wt.% water) at 30 °C. In the study [51], blend HEC/SA (25/75 wt.%) membranes ionically cross-linked with phosphoric acid (PA) were developed for the separation of t-butanol/water mixtures. The influence of parameters such as feed composition, membrane thickness, and permeate pressure on the membrane transport characteristics was studied in ref. [51]. It was shown that the developed HEC/SA membrane had a high potential for separation of azeotropic t-butanol—water mixture at 30 °C, demonstrating high selectivity (99.7 wt.% water in the permeate) and 0.23 kg/(m^2^h) permeation flux. The main disadvantages of the blend HEC/SA membranes for pervaporation dehydration are their low stability in diluted aqueous solutions and low permeability. These parameters could be essentially improved by the change of inner and surface properties of a polymer blend film with its bulk and surface modification, with correct choice of cross-linking agent, and with the development of supported membranes with decreased thickness of selective layer.

To improve physicochemical properties and to acquire tailored transport characteristics of the HEC/SA membranes for pervaporation dehydration of isopropanol various approaches may be applied. For example, the first approach can be the introduction of carbon nanoparticles in HEC/SA blend membranes. The modification of the polymer matrix with carbon nanoparticles, in particular, with water-soluble fullerene derivatives, is one of the perspective and relevant directions for improving the pervaporation performance of polymer membranes demonstrated in earlier studies [59,60,61,62,63]. Especially, the introduction of fullerenol into the polymer matrix leads to a more rigid structure, changes in inner morphology, and greater hydrophilization of the membrane surface, due to the presence of an increased number of polar groups [59,60,61,62,63]. This causes the increased permeability and improves or maintains high selectivity of the membrane. Despite the promise of this direction, a literature review demonstrates that there are no previous studies devoted to the modification of HEC/SA membranes with carbon nanoparticles.

The development of supported membranes with thin selective dense layer based on HEC/SA blend and its composite with fullerenol may be the second approach. One of the most effective methods to increase the membrane permeability is the decreasing of membrane thickness, specifically the development of supported membranes [12,64]. It consists of a thin selective polymer or composite layer on the surface of porous substrate, which provides mechanical strength to the selective layer and does not interfere with mass transfer of components through the membrane. Currently, only the application of supported (composite) membranes is preferred in industry (for example, water treatment, gas separation, etc.) to enhance the robustness and efficiency of the separation process [65,66,67]. A decrease in the thickness of dense polymer membranes from ~20–50 to 0.5–1.5 µm makes them brittle, reducing their mechanical properties, while applying of a thin layer on a stable porous substrate prevents the destruction of this selective layer and ensures its stability. Various filtration porous commercial or in-house substrates based on aromatic polysulfonamide, polysulfone, polyactrinitrile, etc. (consisting of mechanically rigid materials) are usually selected, depending on the separation task, to develop such supported membranes [12,68].

The application of layer-by-layer (Lbl) assembly for the deposition of polyelectrolytes (PEL) on supported membrane surface may be the additional approach for modulating properties of developed supported membranes. The improvement of transport characteristics of membranes may be related to the functionalization and changes in the hydrophilic/hydrophobic properties of the membrane surface, charge overcompensation mechanisms, and surface charge [68,69,70]. The Lbl technique of PEL deposition is a method of sequential deposition of polycations and polyanions on the membrane surface. The properties of membranes can be changed depending on the separation task varying such experimental parameters as applied PEL pairs, the deposited layer thickness, pH of PEL solutions, their ionic strength, etc. [71,72,73]. In the earliest works of our group, it was shown that a correctly selected PEL pair, the applying of several bilayers, and the order of their deposition by Lbl significantly improved the transport characteristics of the membranes based on polyvinyl alcohol for pervaporation dehydration of isopropanol [68,69,74].

Certainly, it should be mentioned that the selection of the optimal cross-linking agent for the membranes and the selection of the optimal ratio of polymers in the membrane matrix are the important tasks to provide the membrane with tailored transport characteristics. In this work, the most commonly used cross-linking agents for SA such as calcium chloride, and phosphoric and citric acids were used. The application of these would allow using the membranes in pervaporation in a wide range of concentrations of water-alcohol mixtures [75,76,77].

Thus, the aim of the present work was to develop novel highly efficient membranes based on HEC/SA blend with improved transport characteristics for pervaporation dehydration of isopropanol using bulk and surface modifications for promising industrial application. Bulk modification was carried out by the introduction of water-soluble fullerene derivative—fullerenol into the HEC/SA membrane matrix with optimal composition (HEC/SA ratio). Fullerenol can act as both a modifier and a cross-linking agent, due to the presence of polar hydroxyl groups causing the changes in inner free volume and surface functionalization of the membrane. The optimal cross-linking method and cross-linking agent (calcium chloride, and phosphoric and citric acids) were selected for the use of HEC/SA membranes in pervaporation separation of diluted solutions. Two types of HEC/SA membrane containing fullerenol were developed: dense and supported on porous polyacrylonitrile (PAN) substrate (to increase the membrane performance). The structural features and physicochemical characteristics of HEC/SA and HEC/SA–fullerenol membranes were investigated by nuclear magnetic resonance (NMR) and Fourier-transform infrared (FTIR) spectroscopies, atomic force (AFM) and scanning electron (SEM) microscopies, thermogravimetric analysis (TGA), contact angle measurements, and swelling experiments. Transport properties of the developed HEC/SA membranes were tested in pervaporation dehydration of isopropanol. The surface modification of supported HEC/SA/fullerenol membrane with optimal properties was carried out by layer-by-layer assembly for the deposition of polyelectrolyte layers to increase the performance of this membrane to facilitate its promising application in industrial dehydration. Thus, the novelty of the work consists of the selection of the membrane component optimal composition, the application of cross-linking agents (mostly used for SA membranes), which are different from the applied for the HEC/SA blend in the literature. Additionally, for the first time, the bulk (the introduction of fullerenol in HEC/SA matrix) and surface (layer-by-layer deposition of polyelectrolytes) modifications of HEC/SA membranes were carried out.

## 2. Materials and Methods

### 2.1. Materials

Hydroxyethyl cellulose (HEC) (Brookfield viscosity of 1% solution 3100 cps, pH of 1% solution 7.1, “Ashland”, Moscow, Russia) and sodium alginate (SA) (viscosity of 90 cps, “BIOPROD” Ltd., St. Petersburg, Russia) were blended in various ratios and applied for the membrane matrix. Water-soluble fullerenol (C_60_(OH)_22–24_) purchased from “Fullerene Technologies” (St. Petersburg, Russia) were used for the modification of HEC/SA membrane. Citric and phosphoric acids, calcium chloride (cross-linking agents), and isopropanol (i-PrOH) (Vekton, St. Petersburg, Russia) were used without additional treatment. The properties of cross-linking agents and isopropanol are presented in Table 1.

Porous membrane based on polyacrylonitrile (PAN, Institute of Physical Organic Chemistry, National Academy of Sciences of Belarus, Minsk, Belarus) was used as a membrane substrate for the development of supported membranes with a thin selective dense layer based on HEC/SA blending. Poly(sodium 4-styrenesulfonate) (PSS) (average MW ~70,000 Da, “Sigma-Aldrich”, St. Petersburg, Russia) was used as polyanion, while poly(allylamine hydrochloride) (PAH) (average MW 50,000 Da, “Sigma-Aldrich”, St. Petersburg, Russia) and SA were used as polycations for the surface modification of supported membranes by Lbl assembly.

### 2.2. Membrane Preparation

#### 2.2.1. Dense Membranes

For the preparation of membranes, the exact amount of HEC powder was dissolved in 1 wt.% SA aqueous solution with the addition of water to obtain 1 wt.% solution of blend polymers HEC/SA. The obtained solution with polymers was dissolved under stirring for 5 h at 45 °C. To select the optimal composition of the membrane, the dense blend membranes were developed varying the composition ratio of HEC and SA—10/90, 30/70, and 50/50 wt.%. Membranes with a higher HEC concentration in the matrix were also prepared and tested in pervaporation experiments. However, it was shown that a higher concentration of HEC led to deterioration of transport performance of the membranes, loss of property reproducibility, and made the membrane brittle. This effect was also observed in the work [51]. The composite of HEC/SA was prepared by the addition of 5 wt.% fullerenol with respect to the HEC/SA mixture with the subsequent active stirring and ultrasonic treatment of the resulting solution. Previous studies demonstrated that for the modification of polymer matrices with fullerenol or other fullerene derivatives, 5 wt.% was the optimal concentration [59,61,62]. Thus, this concentration was selected for the current study. Dense membranes based on HEC/SA and composite HEC/SA/fullerenol (5%) were formed on a Petri dish by solvent evaporation in an oven at 40 °C for 24 h. The thickness of dense membranes measured by the micrometer was 30 ± 5 μm.

#### 2.2.2. Cross-Linking of the Membranes

The developed membranes were cross-linked by immersing the membranes at ambient temperature in different cross-linking agents commonly used for SA such as calcium chloride (CaCl_2_), and phosphoric (PA) and citric (CA) acids: (1) 1.25 wt.% calcium chloride (CaCl_2_) aqueous solution for 10 min, (2) 3.5 vol.% phosphoric acid in isopropanol/water (90/10 wt.%) mixture for 3 h, and (3) 3.5 wt.% citric acid in isopropanol/water (70/30 wt.%) mixture for 3 h [75,76,77,78]. After the cross-linking, membranes were washed with deionized water to eliminate the excess of the cross-linking agent.

#### 2.2.3. Supported Membranes

To increase the permeability of the best developed membranes, supported membranes were prepared. These membranes consisted of the thin dense layer based on HEC/SA (30/70 wt.%) or HEC/SA (30/70 wt.%) with fullerenol (5 wt.% with the respect to the HEC/SA blend) with the thickness ~1 μm, deposited onto the surface of a porous membrane (substrate) based on polyacrylonitrile (PAN). The choice of PAN substrate was justified by its hydrophilic surface properties and porosity structure, which ensured good adhesion of the thin selective polymer layer (~1 µm thickness) to the substrate providing reliable mechanical support and insignificantly affecting the mass transfer of components through the membrane. Then the supported membranes were dried at ambient temperature for 24 h to remove the solvent and cross-linked with calcium chloride as previously described. The solutions for the preparation of a thin dense layer were prepared according to the procedure described for the dense membranes. Good adhesion of a thin selective layer on the porous PAN substrate was confirmed by scanning electron microscopy (SEM). Cross-sectional SEM micrographs of supported membranes before and after pervaporation demonstrated the presence of two uniform layers: (1) the upper dense layer based on SA/HEC and its composite without defects, and (2) the bottom layer of porous PAN substrate.

The designations of the membranes based on HEC/SA (30/70 wt.%) and the conditions of their preparation used are presented in Table 2. The number “5” after a dash indicates the content of fullerenol (5 wt.%) in the HEC/SA membranes, the PAN substrate is indicated through a slash for the supported membranes, cross-linking reagents are presented as abbreviations CaCl_2_, PA, and CA in the superscript.

#### 2.2.4. Modification with Layer-by-Layer (Lbl) Technique

The surface modification of the developed supported HEC/SA-5/PAN^CaCl2^ membrane was carried out by layer-by-layer technique using the Xdip-MV1 robotic immersion (dipping) coating system (“PROMENERGOLAB” Ltd., Moscow, Russia). PAH, SA, and PSS were used as polyelectrolytes (PEL) in the form of solutions with the concentration of 10^−2^ mol/L. The membrane was fixed in the device and alternately immersed in polyelectrolyte solutions for 10 min, and washed in water between them [68]. First, a membrane is immersed in polyanion solution of the PSS as it is based on the SA, which itself is a polyelectrolyte with positive surface charge, and then it was thoroughly washed with water. Thereafter, the membrane is immersed in polycation solution of PAH or SA with subsequent washing of the membrane. Thus, one PEL bilayer was formed on the membrane surface. In this work, the formation of five bilayers were shown to be the optimal number of cycles (depositions of PEL), resulting in the improved membrane performance.

### 2.3. Pervaporation

The transport properties of the developed membranes were evaluated in pervaporation dehydration of isopropanol in a steady-state regime with stirring, at ambient temperature (22 °C) and with a downstream pressure of 10^−1^ mm Hg. The pervaporation setup was described in detail in the work [63]. The composition of the permeate and the feed was determined using a Chromatek Crystal 5000.2 chromatograph (Chromatec, Nizhny Novgorod, Russia) with a thermal conductivity detector and a “Hayesep R” column. The transport parameters of membranes were calculated and demonstrated in terms of permeation flux, component permeances (P/l), water content in the permeate, and pervaporation separation index (PSI).

The permeation flux (J) was calculated by the following equation [79]:

(1) $$J\;=\;\frac{W}{A\times t}$$

where *W* is the weight of permeate (kg), *A* is the membrane area (m^2^), and *t* is the time of permeate collection (h).

The pervaporation separation index (PSI) is well accepted to evaluate the overall pervaporation performance. It is defined as a composite parameter of permeation flux and separation factor, which can serve as a measure of the pervaporation separation ability of a membrane for a binary mixture under the specified experimental conditions [80]. The pervaporation separation index (PSI) was calculated by the following equation [81]:

(2) $$PSI\;=\;J\times \left(\beta -1\right)$$

where *J* is the permeation flux, *β* is the separation factor, calculated as [82]:

(3) β = yiyjxixj

where *y_i_* and *y_j_*, *x_i_* and *x_j_* are the fractions of i and j components in the permeate and the feed, respectively.

The permeance P/l was calculated by the following equation [82]:

(4) $$\frac{P}{l}\;=\;\frac{j_{i}}{p_{i_{f}}-p_{i_{p}}}$$

where *j_i_* is the partial flux of *i* component, $p_{i_{f}}$ and $p_{i_{p}}$ are vapor pressures of *i* component in the feed and the permeate, respectively, *l* is the membrane thickness. The permeance was expressed in gas permeation units GPU (1 GPU = 1 × 10^−6^ cm^3^ (STP)/cm^2^ s cm Hg).

Each measurement was carried out at least three times, and the average value was taken for later analysis. The mean accuracy for the transport parameters was as follows: ±0.5% for water content in the permeate; ±1% for permeation flux for the dense and supported membranes, respectively.

### 2.4. Membrane Characterization

Structural changes of the developed membranes were studied by Fourier-transform infrared spectroscopy (FTIR) using an IRAffinity-1S spectrometer (Shimadzu, St. Petersburg, Russia) in the range of 650–4000 cm^−1^ at 25 °C and a resolution of 2 cm^−1^.

The developed membranes were also studied by nuclear magnetic resonance (NMR) using a Bruker Avance III 400 WB spectrometer (Bruker, Ettlingen, Germany) with a 4-mm CP/MAS probe and 100.64 MHz Larmor frequency for ^13^C nuclei. Tetramethylsilane (TMS) was used as an external reference for ^13^C nuclei. {^1^H}^13^C CP/MAS NMR experiment conditions were as follows: 8192 scans, 5 s relaxation, and 2 ms contact time.

The cross-sectional and surface morphology of the membranes was investigated by scanning electron microscopy (SEM) using a Zeiss Merlin SEM (Carl Zeiss SMT, Oberhochen, Germany). The experiment conditions were as follows: 1 kV low accelerating voltage and 100 pA electron beam current.

The surface topography of the developed membranes was studied by atomic force microscopy (AFM) using an NT-MDT NTegra Maximus atomic force microscope (NT-MDT Spectrum Instruments, Moscow, Russia) in tapping mode and standard silicon cantilevers (15 N·m^−1^ rigidity).

The thermochemical properties of the membranes were investigated by thermogravimetric analysis (TGA) using a Thermobalance TG 209 F1 Libra (Netzsch, Leuna, Germany). The experiment conditions were as follows: the weight of the samples of ~2 mg, 30–600 °C the temperature range, 10 °C/min the heating speed; the measurements under argon atmosphere.

The equilibrium swelling degree of the developed membranes was evaluated in isopropanol, water, and azeotropic water-isopropanol (12/88 wt.%) mixture by a gravimetric method at 22 °C. The membranes of known weight were immersed in weighing bottles with solvents and azeotropic mixture, and weighed day after day until the constant weight. Then the membranes were dried at 40 °C for 24 h for the evaluation of solvent desorbing from the membranes.

The swelling degree (S) of membranes was calculated by the following equation:

(5) $$S\;=\;\frac{m_{o}-m_{p}}{m_{p}}$$

where *m_o_* is the weight of swollen membrane (including the weight of polymer blending and weight of solvent inside of membrane) (g), *m_p_* is the membrane weight after the swelling experiments and drying at 40 °C.

Contact angles of water were measured on both sides of dense membranes and on the selective layer of supported membranes by the sessile drop method using Goniometer LK-1 device (“NPK Open Science” Ltd., Krasnogorsk, Russia). The obtained results were processed in the “DropShape” software.

## 3. Results

This part is divided into several sections. Section 3.1 is dedicated to transport properties in the pervaporation dehydration of isopropanol of the dense untreated (Section 3.1.1) and cross-linked (Section 3.1.2) membranes with different blend HEC/SA compositions and/or with fullerenol modification. Further, in Section 3.2, the explanation of the obtained transport characteristics using membrane structure and physicochemical properties studied by the different methods of analysis (FTIR and NMR spectroscopy, SEM, AFM, TGA, contact angle, and swelling degree measurements) is presented. The study of the structural characteristics of the dense membranes was carried out to exclude the influence of porous substrate and defects of the membranes. In Section 3.3, the results on transport and structural properties of cross-linked supported membranes are presented. The supported membranes were developed to increase the performance of cross-linked dense membranes (Section 3.3.1). The additional improvement of transport characteristics of supported membranes was achieved by layer-by-layer assembly with the deposition of the polyelectrolyte layers (Section 3.3.2). The presence and stability of polyelectrolyte layers on the membrane surface was confirmed by SEM, AFM, and contact angle measurements before and after pervaporation. The last Section 3.4 is devoted to the comparison of the performance of the developed membranes with membranes described in the literature under similar experimental conditions.

### 3.1. Pervaporation Using Dense Membranes

#### 3.1.1. Study of the Untreated Membranes

**Figure 1:**
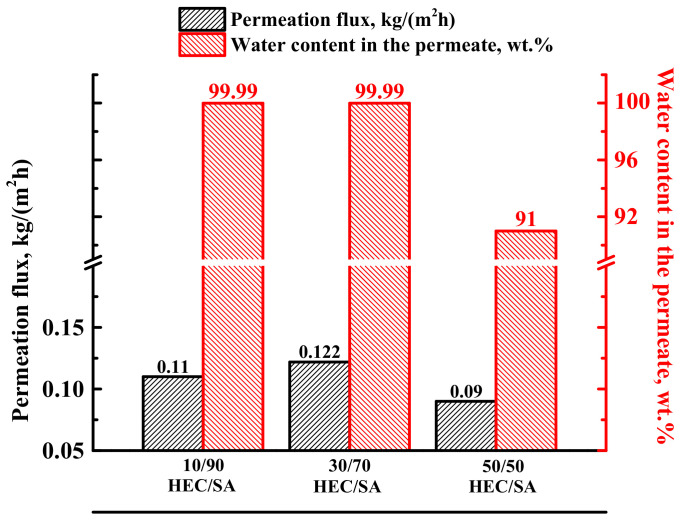
The permeation flux and water content in the permeate of the dense blend HEC/SA membranes in pervaporation separation of the azeotropic water (12 wt.%)—isopropanol (88 wt.%) mixture at 22 °C.

To evaluate the effect of the HEC/SA composition on the transport properties in pervaporation dehydration, blend membranes were prepared by mixing of HEC and SA in different ratios: 10/90, 30/70, and 50/50 wt.%. It is also worth noting that a further increase in the HEC content in the mixture made the membrane brittle [51]. Transport characteristics of the developed dense blend HEC/SA membranes were tested in pervaporation for the separation of the azeotropic water (12 wt.%)—isopropanol (88 wt.%) mixture (Figure 1).

It was obtained that the increase of HEC content in SA matrix up to 30% led to a slight increase in permeation flux, maintaining a high level of selectivity to water (99.99 wt.% in the permeate). However, the further increase of HEC content up to 50 wt.% caused the decrease of both parameters—permeation flux and water content in the permeate (Figure 1). The same effect of decreasing permeation flux with high HEC content in the membrane matrix was observed in [52]. Based on the data received, the HEC/SA (30/70 wt.%) membrane was found to have the optimal transport properties in pervaporation separation of the azeotropic water–isopropanol mixture. This was due to the fact that in this polymer blend composition, the polar groups did not overlap and were evenly distributed in space without interfering with each other. The intermolecular interactions of HEC/SA blend were confirmed by FTIR and NMR spectroscopies (Section 3.2.1 Study of structural characteristics). In order to improve permeability of this membrane, its modification with 5 wt.% fullerenol was carried out. Transport properties of the HEC/SA-5 (with 5 wt.% fullerenol) membrane were also evaluated in pervaporation separation of the azeotropic water (12 wt.%)–isopropanol (88 wt.%) mixture for the comparison with the pristine HEC/SA membrane (Table 3).

The modification of the HEC/SA matrix with 5 wt.% fullerenol led to the increase of permeation flux by 17% and under high selectivity to water preserved (99.99 wt.% in the permeate, Table 3). Several factors may explain the results obtained: (i) fullerenol significantly changed the inner and surface morphology of the HEC/SA membrane, leading to the rise of membrane surface roughness and more homogeneous dispersion of the polymers in the blend (SEM and AFM data in Section 3.2.1). Fullerenol particles on the membrane surface and increased surface roughness led to additional sorption centers and, consequently, to an increase in membrane permeability according to the pervaporation “solution-diffusion” mechanism. Fullerenol also acts as a cross-linking agent due to the formation of hydrogen bonds between its molecules and polymer chains (FTIR data described in Section 3.2.1). Cross-linking reduces the inner space between polymer chains and maintaining high selectivity of the membrane. Thus, fullerenol functions as both a modifier and a cross-linking agent, leading to an improvement in the transport characteristics of HEC/SA membrane. However, for the successful implementation of this modified HEC/SA membrane (HEC/SA-5) in industrial processes, it is necessary to select the optimal cross-linking method to carry out pervaporation experiments in the wide concentration range. The cross-linking experiments are presented in the next section.

#### 3.1.2. Study of Cross-Linked Membranes

**Figure 2:**
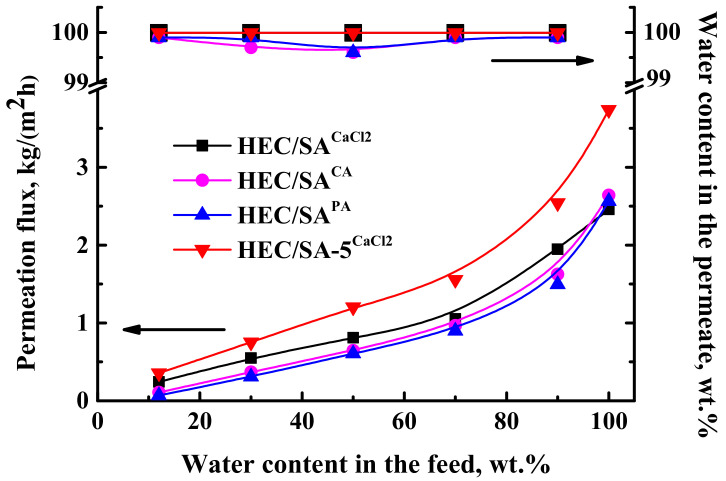
The dependence of permeation flux and water content in the permeate on water content in the feed for the HEC/SA membranes cross-linked with calcium chloride (CaCl_2_), and phosphoric (PA) and citric (CA) acids, and HEC/SA-5 membrane cross-linked with calcium chloride (CaCl_2_) in pervaporation dehydration of isopropanol (12–100 wt.% water) at 22 °C.

For the application of the developed dense HEC/SA and HEC/SA-5 membranes in pervaporation dehydration of isopropanol in a wide concentration range (12–100 wt.% water), a chemical cross-linking of membranes based on pristine HEC/SA blend with various agents such as CaCl_2_, PA, and CA was carried out [75,76]. Transport properties of the cross-linked dense HEC/SA membranes were tested in pervaporation dehydration of isopropanol (12–100 wt.% water). The data are presented in Figure 2.

The data in Figure 2 demonstrated that the application of cross-linking methods allowed using HEC/SA membranes for the separation of isopropanol-water mixture in the whole concentration range, including pure water. The cross-linking with PA led to the least permeation flux values compared to others, whereas the cross-linking with CaCl_2_ resulted in the highest permeation flux of the HEC/SA membrane. It is also worth noting that the selectivity to water for the HEC/SA^PA^ and HEC/SA^CA^ membranes is reduced compared to the cross-linked HEC/SA^CaCl2^ membrane, for which water content in the permeate is constant at 99.99 wt.% (Figure 2). The increased permeation flux with the highest level of selectivity for the HEC/SA^CaCl2^ membrane may be explained by changes in structure and physicochemical properties. The cross-linkage and the intermolecular interactions of the HEC/SA blend were confirmed by FTIR and NMR spectroscopies (Section 3.2.1) and by swelling experiments in the pure water (Section 3.2.2). The cross-linking with CaCl_2_ of the HEC/SA membrane organized the polymer chains into electronegative cavities in the shape of “egg box” (confirmed by FTIR data in Section 3.2.1), promoting increased water penetration and retention of alcohol molecules by ionic interactions, which contributed to maintaining a high level of selectivity [78]. Additionally, the HEC/SA^CaCl2^ membrane had the roughest surface (confirmed by AFM data in Section 3.2.1) and an increased hydrophilic matrix character (confirmed by the least contact angle of water and the highest swelling degree in Section 3.2.2) among the membranes, cross-linked with citric or phosphoric acids. These contributed to an increased number of sorption centers on the membrane surface and high affinity to water leading to the highest permeation flux. Thus, the cross-linking method of the HEC/SA membrane immersing in 1.25 wt.% CaCl_2_ aqueous solution for 10 min was selected as optimal.

For the improving of the HEC/SA^CaCl2^ membrane permeability, the membrane was modified with 5 wt.% fullerenol. The transport characteristics of the modified HEC/SA-5^CaCl2^ membrane were also presented in Figure 2. It was demonstrated that the HEC/SA-5^CaCl2^ membrane had increased permeation flux by 30–50%, compared to the HEC/SA^CaCl2^ membrane, with the same level of selectivity (99.99 wt.% water in the permeate). These changes are associated with the effect of fullerenol on the cross-linking process. Fullerenol decreased the cross-linking effect of CaCl_2_ reducing the number of linked HEC/SA blocks (confirmed by FTIR and NMR data). This led to the more ordered and evenly roughened membrane structure with increased surface roughness (confirmed by SEM and AFM data), and surface hydrophilization (data of contact angle measurements), compared to the unmodified cross-linked HEC/SA membrane resulting in increased membrane permeability [63]. At the same time, it acted as a cross-linking agent due to bonding with polymer chains preventing a decrease in membrane selectivity to water. This effect was also confirmed by FTIR and NMR (Section 3.2.1). Thus, based on the results obtained, the HEC/SA-5^CaCl2^ membrane had the best transport characteristics in pervaporation dehydration of isopropanol compared to the untreated HEC/SA, HEC/SA-5, and cross-linked HEC/SA^CaCl2^ membranes.

To explain the obtained dependences of the transport characteristics and the mass transfer of low molecular weight penetrants through membranes, the structural features and physicochemical properties of the dense membranes were studied by various analytical methods and presented in the next section.

### 3.2. Investigation of Dense Membranes

#### 3.2.1. Study of Structural Characteristics

**Figure 3:**
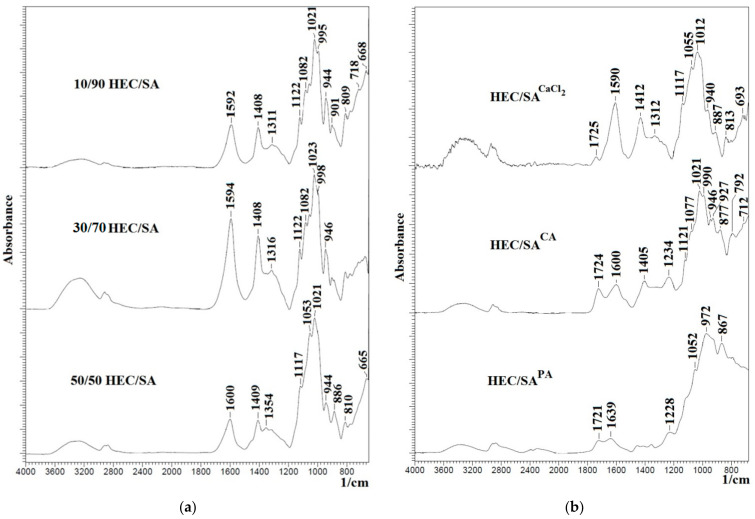
The Fourier-transform infrared (FTIR) spectra for (**a**) the HEC/SA membranes with different polymer weight ratios (10/90, 30/70, and 50/50 wt.%) and (**b**) the cross-linked HEC/SA (30/70) membrane with calcium chloride (CaCl_2_) and phosphoric (PA) and citric (CA) acids.

The structure of developed blend membranes was investigated by FTIR and NMR spectroscopies, SEM and AFM microscopies. FTIR spectra for the blend HEC/SA membranes with different polymer weight ratio (10/90, 30/70, and 50/50 wt.%) and for the HEC/SA membrane cross-linked with various agents are presented in Figure 3.

FTIR spectra in Figure 3a demonstrate the presence of characteristics peaks of HEC and SA polymers: peaks at ~3280, 1408, in the range at 2800–3000, 1020–1095 cm^−1^ corresponding to the stretching of -OH group, the –C-O-C-stretching vibrations, absorption bands of the –CH and –CH_2_ groups, and the stretching of aliphatic C–H and C–O bonds, respectively [76,83,84,85,86]. However, when polymers are blended in different ratios, some changes in the IR spectra are observed. The broadening of the peaks at 1592 and 1311 cm^−1^ and its shift to 1594 and 1600 cm^−1^, 1316 and 1354 cm^−1^, respectively, may be related to the superposition of the peaks of SA and HEC. It is also worth noting that in the IR spectra of the HEC/SA membrane (with 30/70 wt.% composition), the peaks at ~3280, 1594, 1408, 1316, and 1122 cm^−1^ have the highest intensity in comparison with the HEC/SA (10/90) and HEC/SA (50/50) membranes. This effect may be related to the fact that at this ratio, an optimal degree of homogeneity of the polymer distribution of the system is achieved. The blended system is characterized by an ordered distribution of the mixture components causing the uniform dispersion of polar groups of polymers HEC and SA in the polymer blend.

The spectra of the HEC/SA membranes cross-linked with various agents are presented in Figure 3b. It was shown that the cross-linking with CaCl_2_ of the HEC/SA membrane led to maintaining the peak intensity at ~3280 cm^−1^ of hydroxyl groups, the peak shift from 1594, 1408, and 1316 cm^−1^ to 1590, 1412, and 1312 cm^−1^, respectively, and the decrease of intensity of these peaks. These changes indicate the ionic interaction of HEC and SA with Ca^2+^ forming the “egg box” structure, causing the cross-linking of polymer chains [63,75,76]. For the HEC/SA^CA^ membrane, the broadening of the peak at 1600 cm^−1^ (C=O stretching) and the presence of peak at 1234 cm^−1^ (corresponding to ether linkage C-O-C between glucose rings in HEC backbone chains) indicate the cross-linking of the HEC/SA membrane with CA [87]. In the spectrum of the HEC/SA^PA^ membrane, the presence of peaks in the range of 1300–1400 cm^−1^ (the specific absorbance of the −P−O−C-bond) and at 972 cm^−1^ (corresponding to P=O group) confirmed the cross-linking of HEC/SA by PA [54,55]. In addition, it should be noted that for all cross-linked HEC/SA^CaCl2^, HEC/SA^CA^, and HEC/SA^PA^ membranes, there are peaks at 1725, 1724, and 1721 cm^−1^, respectively, assigned to the formation of ester bond. The presence of these peaks confirms the cross-linking reactions between CA, PA hydroxyl, SA carboxyl, and HEC hydroxyl groups [54,55,87].

**Figure 4:**
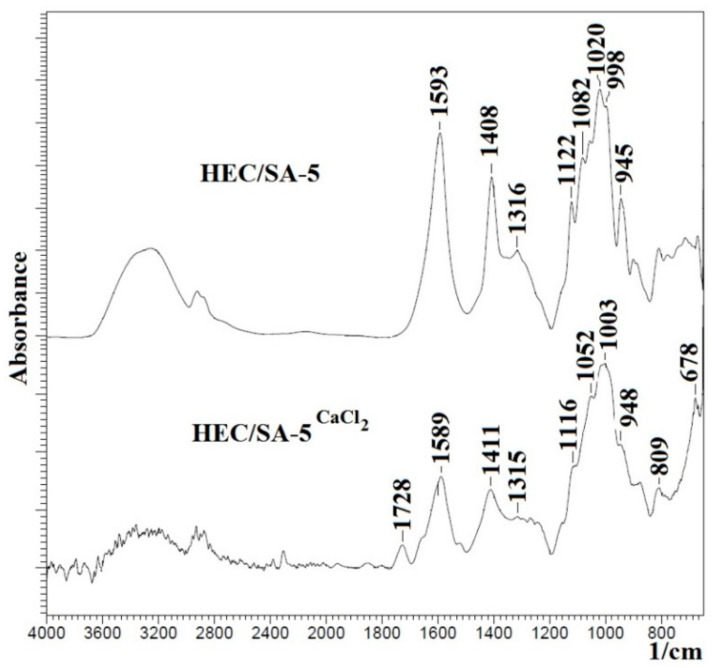
The FTIR spectra for HEC/SA-5 and cross-linked HEC/SA-5^CaCl2^ membranes.

FTIR spectra for the blend HEC/SA membrane, modified with 5 wt.% fullerenol or/and cross-linked with CaCl_2_ (HEC/SA-5 and HEC/SA-5^CaCl2^ membranes), are presented in Figure 4.

It was shown that the FTIR spectra for the HEC/SA-5 and HEC/SA membranes actually did not differ (Figure 3a and Figure 4), except for an increase in the peak at ~3280 cm^−1^ for the modified HEC/SA-5 membrane. This indicates an increase in hydroxyl group numbers in the membrane matrix leading to the improving of the membrane’s hydrophilicity. The comparison of FTIR spectra of the cross-linked HEC/SA^CaCl2^ and HEC/SA-5^CaCl2^ membranes demonstrates that there are no significant changes in the structure of the 5 wt.% fullerenol modified membrane (Figure 3b and Figure 4). However, the decrease of peak intensity at ~3280 cm^−1^, 1589, and 1411 cm^−1^ for the HEC/SA-5^CaCl2^ membrane may be related to the leveling effect of fullerenol on the cross-linking with calcium chloride, which leads to the reduced cross-linking degree of polymer chains and the reduced linked polymer block number [63]. Namely, fullerenol modification led to the decrease of “crystallized” phase of the cross-linked membrane and the formation of a more amorphous structure (confirmed by NMR data below). The cross-linking of the HEC/SA-5 membrane with CaCl_2_ led to the following changes: the decrease of the peak intensity at ~3280 cm^−1^ of -OH groups, the decrease of peak intensities and their shift from 1593, 1408, and 1020 cm^−1^ to 1589, 1412, and 1003 cm^−1^, respectively (Figure 4). These changes demonstrate that simultaneous application of fullerenol and calcium chloride to cross-link the membrane exhibit a synergetic effect as compared to individual fullerenol, which ensures the membrane’s stability in diluted solutions during pervaporation.

**Figure 5:**
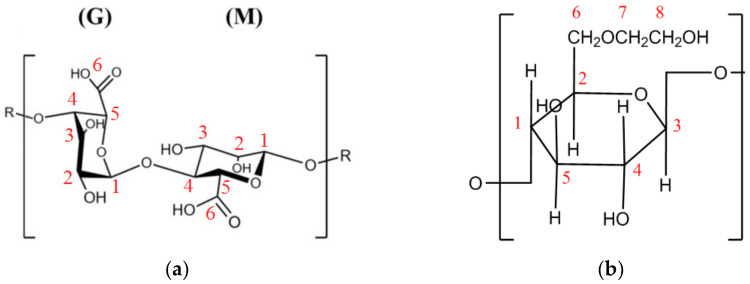
Schematic representation of (**a**) alginate with the blocks of munnuronate (M) and guluronate (G), and (**b**) hydroxyethyl cellulose.

Structural features of the developed membranes were also investigated by NMR spectroscopy. Schematic representation of alginate and HEC polymers with indication of the position number of corresponding carbon atoms is presented in Figure 5 to simplify the presentation of NMR data.

**Figure 6:**
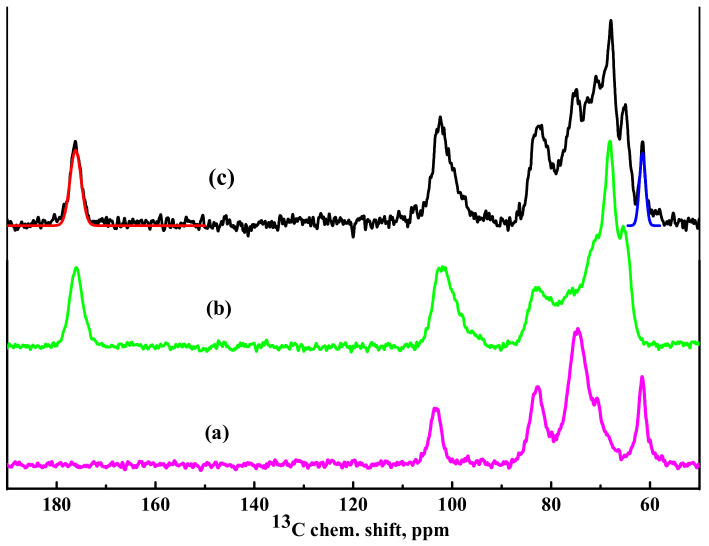
^13^C nuclear magnetic resonance (NMR) spectra of (**a**) HEC, (**b**) SA, and (**c**) HEC/SA membranes. The approximations of the spectral components corresponding to the carboxyl group of SA (about 176 ppm, red line) and the methanol group of HEC (about 61.5 ppm, blue line) are demonstrated in spectrum (**c**).

^13^C NMR spectra of HEC, SA, and HEC/SA membranes are demonstrated in Figure 6.

It should be noted that in the spectrum for HEC membrane (Figure 6), there are no peaks indicating the bonding of carbon atoms in position 4 to any functional groups of molecular structures other than -OH. It was shown that the NMR spectrum of the membrane based on blend of polymers (HEC/SA with 30/70 wt.% composition) (Figure 6) is practically a simple addition of two spectra of SA and HEC polymers (Figure 6). This indicates that in the blend HEC/SA membrane there are no structural changes in polymers related to chemical interactions. The approximating curves, corresponding to the carbon atoms in the methanol group of HEC (about 61.5 ppm) and carboxyl group of SA (about 176 ppm), are presented in Figure 6. The ratio of the relative integral intensities of these spectral components (-CH_2_OH)/(-COOH) is equal to 34/66, which is in line with the specified ratio of polymers used for the membrane’s preparation.

**Figure 7:**
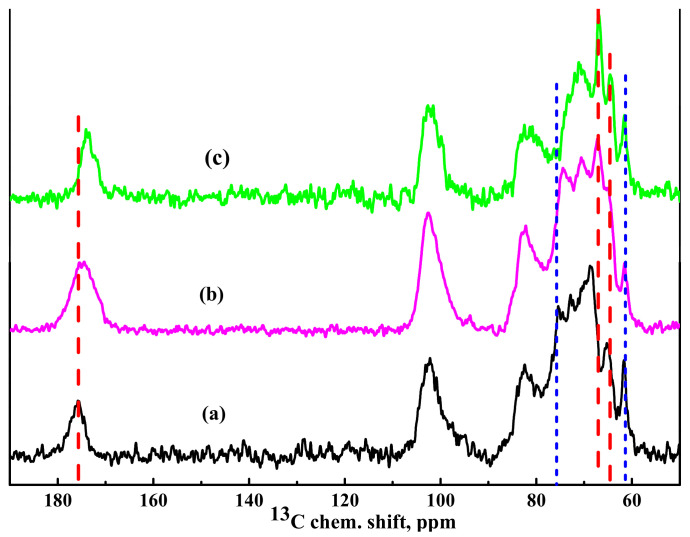
^13^C NMR spectra of the cross-linked (**a**) HEC/SA^CaCl2^, (**b**) HEC/SA^CA^, and (**c**) HEC/SA^PA^ membranes.

^13^C NMR spectra of the HEC/SA (30/70) membranes cross-linked with CaCl_2_, PA, and CA are presented in Figure 7.

It was demonstrated that cross-linking of the HEC/SA membrane with CaCl_2_ led to a slight broadening of the line corresponding to carbon atoms in the carboxyl groups of the SA. It resulted in an insignificant shift of the peaks corresponding to carbon atoms in the G5 positions (about 68.5 ppm) associated with this carboxyl group and in the G2 positions (about 65.3 ppm) due to their participation in the cross-linking mechanism (Figure 7a). At the same time, there is no significant influence on the peaks of other functional groups.

The cross-linking with CA of the HEC/SA membrane led to a significant broadening of the line corresponding to the carbon atoms of SA carboxyl groups, which could be partially explained by the presence of an unresolved signal from the remaining CA molecules in the membrane matrix (Figure 7b). In addition, an insignificant shift of the peak corresponding to carbon atoms in the G2 positions of SA was observed. It could be associated with the formation of weak bonds of the hydroxyl groups with CA molecules during the cross-linking of the membranes [76]. The presence of PA in the HEC/SA membrane manifested in the line broadening in the NMR spectrum (Figure 7c), which corresponded to the carbon atoms in the SA carboxyl groups, and in its significant shift. The influence of PA cross-linking on the rest of the spectrally resolved components of the SA spectrum was practically not observed. At the same time, there was a slight shift of the peak attributed to HEC carbon atoms in the 8 position, as well as a cardinal shift of the unresolved line. The unresolved line corresponded to HEC carbon atoms in the 4, 5, 7 positions. The NMR data and the obtained changes confirmed the interaction of HEC/SA with CaCl_2_, PA, and CA and cross-linking of polymer chains in the membrane.

**Figure 8:**
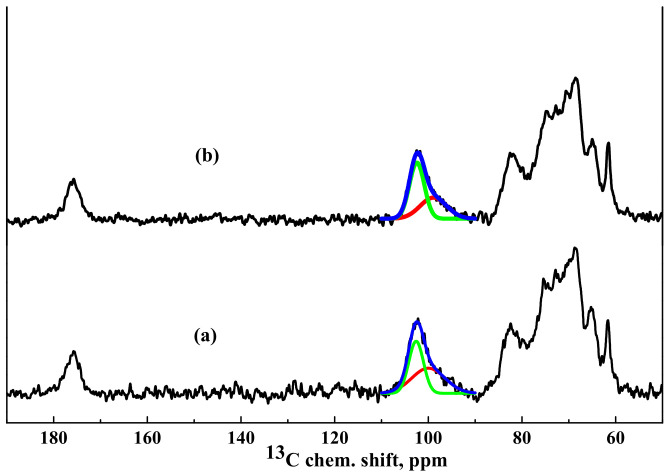
^13^C NMR spectra of cross-linked (**a**) HEC/SA^CaCl2^ and (**b**) HEC/SA-5^CaCl2^ membranes.

^13^C NMR spectra of the HEC/SA^CaCl2^ and HEC/SA-5^CaCl2^ membranes are presented in Figure 8.

It was demonstrated that NMR spectra of the HEC/SA and HEC/SA-5 membranes had no significant differences (Figure 6), except the shift of the peak in the region of 82 ppm (corresponding to the carbon atom in 4 position for SA and in 1 position for HEC) to a greater value of the chemical shift during modification with fullerenol, which indicated changes in the bond lengths in polymer structures. The spectra of the HEC/SA^CaCl2^ and HEC/SA-5^CaCl2^ membranes practically coincide. However, analyzing the unresolved spectral line with a maximum at ~102.5 ppm, corresponding to SA carbon atoms at the G1 and M1 positions and to HEC carbon atoms in the 3 position, it could be seen that the ratio of the two components forming this spectral line changed. In the spectrum of the HEC/SA^CaCl2^ membrane, the component (about 100 ppm) attributed to the “crystallized” phase is equal to 52% of the total integrated intensity of the described unresolved line (Figure 8a). The modification of this membrane with 5 wt.% fullerenol reduced this component to 40% (Figure 8b). This indicates that fullerenol in the membrane led to the formation of a more amorphous structure in the blend membrane and the decrease of cross-linking effect on the HEC/SA matrix caused the increased permeability of the modified membrane (Figure 2).

**Figure 9:**
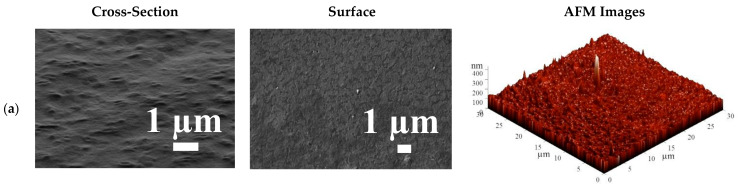

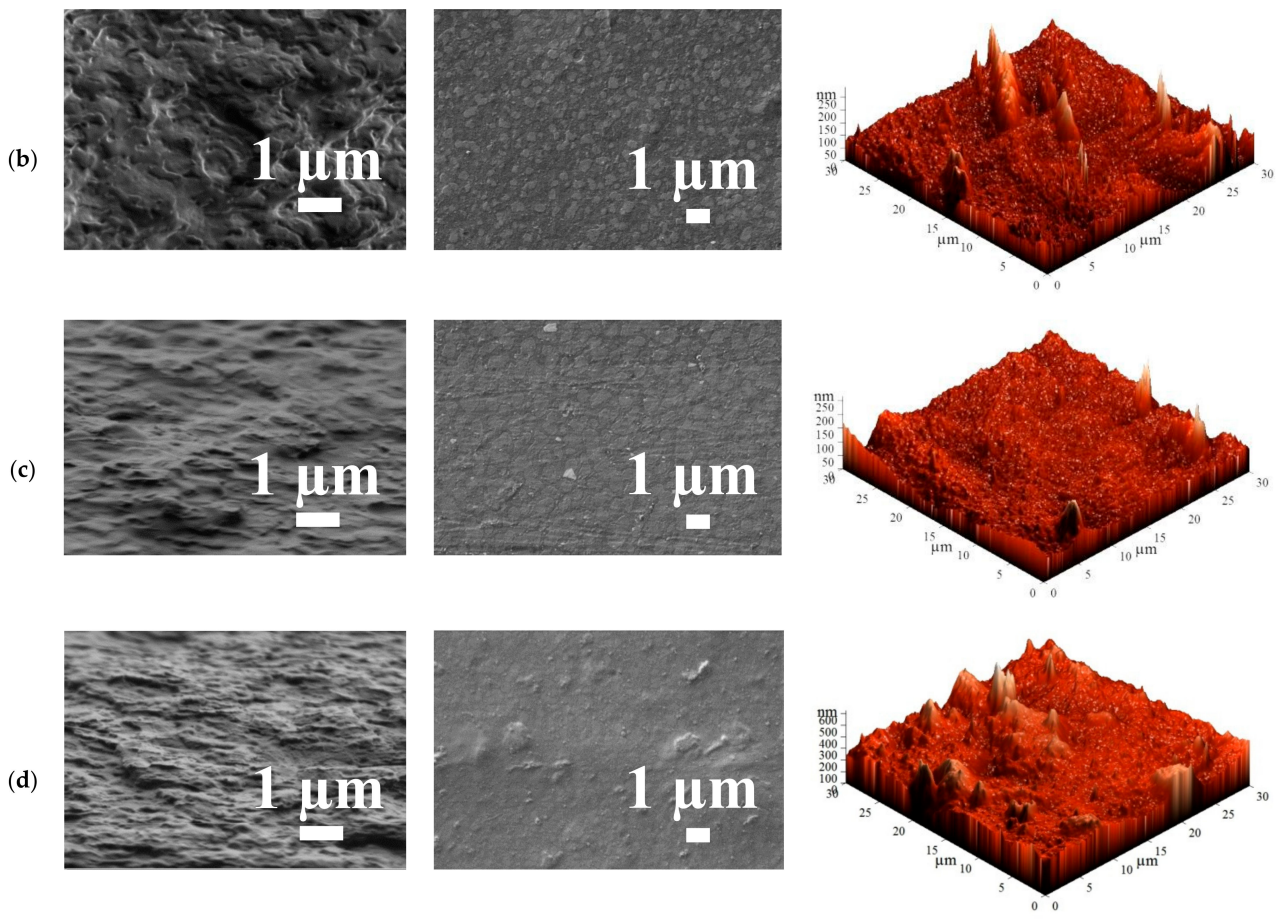
The cross-sectional and surface scanning electron microscopy (SEM) micrographs and atomic force microscopy (AFM) images of the HEC/SA membranes with different polymer weight ratios: (**a**) 10/90, (**b**) 30/70, and (**c**) 50/50 wt.%, and (**d**) HEC/SA-5 membrane.

The morphology of the inner structure and surface of the developed membranes were investigated by SEM and AFM methods. The surface and cross-sectional SEM micrographs and AFM images (scan size of 30 × 30 μm) for membranes with different HEC/SA weight ratio (10/90, 30/70, and 50/50 wt.%) and for the HEC/SA-5 membrane (consisting of 30/70 HEC/SA blend modified with 5 wt.% fullerenol) are presented in Figure 9.

The SEM micrographs demonstrated that 30/70 wt.% of the HEC/SA composition led to the most rough and tortuous structure of the cross-section for the blend membrane in comparison with the HEC/SA membranes with 10/90 and 50/50 wt.% polymer ratios. On the SEM surface micrographs, the division of HEC and SA polymers is observed, namely, the inclusion of HEC polymer, which is quite uniform especially for the HEC/SA membrane with 30/70 wt.% ratio. The SEM micrographs of the HEC/SA-5 membrane demonstrated that the introduction of fullerenol into the HEC/SA matrix led to a leveling of the cross-sectional structure of the HEC/SA-5 membrane and more homogeneous membrane surface (the absence of clear outlines of HEC inclusions in SA). Moreover, the particles of fullerenol on the surface of the HEC/SA-5 membrane were observed, which could act as additional sorption centers, leading to an increased permeability of the modified membrane (Table 3).

**Figure 10:**
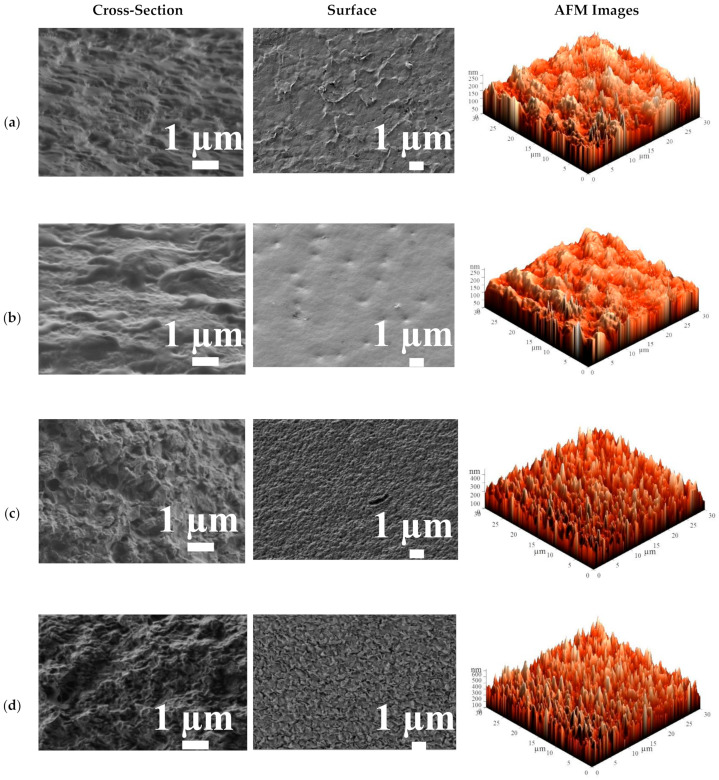
The cross-sectional and surface SEM micrographs and AFM images of the cross-linked HEC/SA membranes: (**a**) HEC/SA^CA^, (**b**) HEC/SA^PA^, (**c**) HEC/SA^CaCl2^, and (**d**) HEC/SA-5^CaCl2^ membranes.

The surface and cross-sectional SEM micrographs and AFM images (scan size of 30 × 30 μm) for the cross-linked HEC/SA membrane with calcium chloride (CaCl_2_), and phosphoric (PA) and citric (CA) acids, and for the HEC/SA-5^CaCl2^ membrane are presented in Figure 10.

The cross-linking with CA, PA, and CaCl_2_ led to the various changes in the HEC/SA membrane morphology. The cross-sectional SEM micrographs demonstrated that the application of CA and PA led to a rough structure of a cross-section with smoother and more uniform transitions (Figure 10a,b), compared to the uncross-linked HEC/SA membrane (Figure 9b). At the same time, the use of CaCl_2_ for the HEC/SA membrane caused aggravated roughness and enhances plastic deformation of the membrane cross-section (Figure 10c) [63]. Based on surface SEM micrographs, the cross-linked HEC/SA^PA^ membrane had the smoother and more homogeneous surface compared to the uncross-linked HEC/SA membrane, due to the effect of stronger cross-linking with PA, which was susceptible to reduced membrane permeability (Figure 2). At the same time, there were significant plastic deformations on the surface of the HEC/SA^CA^ and HEC/SA^CaCl2^ membranes (Figure 10a,c), resulting in the appearance of more sorption centers. This caused the increased permeation flux of membranes. However, the surface SEM micrograph of the HEC/SA^CaCl2^ membrane demonstrated more uniform and evenly distributed roughness over the entire surface compared to the HEC/SA^CA^ membrane, which contributes to more reproducible transport characteristics.

SEM micrographs demonstrated that the cross-linking with CaCl_2_ of HEC/SA and HEC/SA-5 membranes led to a coarser structure of the membrane cross-section and surface compared to the untreated (without additional treatment) membranes (Figure 9b,d). The same effect was observed for the SA and SA/fullerenol (5%) membranes in [63]. The cross-linked modified membrane (HEC/SA-5^CaCl2^) had more structured and evenly roughened cross-sectional and surface structure compared to cross-linked HEC/SA membrane (HEC/SA^CaCl2^) due to the modification with fullerenol. This change in inner structure significantly caused an increase in the performance of the HEC/SA-5^CaCl2^ membrane (Figure 2).

Based on the presented AFM images (Figure 9 and Figure 10), the surface roughness parameters of developed membranes were calculated as average (R_a_) and root-mean-squared (R_q_) surface roughness (Table 4). The calculation of the surface roughness of membranes is necessary since this parameter significantly affects the first stage of the mass transfer mechanism in pervaporation: faster sorption of the feed components on the membrane surface leads to the increase of membrane permeability.

The data in Table 4 demonstrate that the HEC/SA membrane (with 30/70 wt.% composition) has the highest surface parameters (R_a_ = 17 nm, R_q_ = 24.9 nm) among the untreated membranes without fullerenol modification (HEC/SA (10/90) and HEC/SA (50/50)). This may be related to the ordered distribution of the HEC and SA polymers in the membrane matrix (confirmed by FTIR data, Figure 3). The modification of this membrane with 5 wt.% fullerenol led to the increase of surface parameters R_a_ to 32.9 and R_q_ to 46.1 nm, causing the improved permeability compared to the HEC/SA membrane (with 30/70 wt.% composition, Table 3). The application of cross-linking with various agents for the HEC/SA (30/70) membrane also led to the increased surface roughness, which increased in the following sequence: HEC/SA (30/70) < HEC/SA^PA^ < HEC/SA^CA^ < HEC/SA^CaCl2^ membranes. The HEC/SA-5^CaCl2^ membrane had the highest surface roughness parameters compared with all developed membranes, which provided the highest permeability of this membrane in the pervaporation of isopropanol dehydration (Figure 2). All data on surface roughness parameters were in agreement with FTIR, SEM, and pervaporation experiments.

#### 3.2.2. Study of Physicochemical Properties

**Figure 11:**
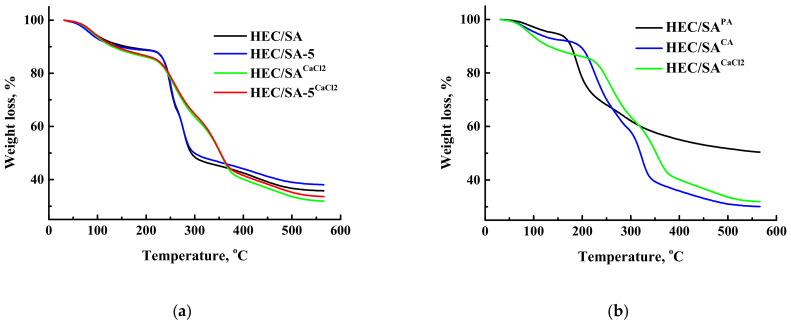
TG curves for (**a**) the untreated and cross-linked HEC/SA and HEC/SA/fullerenol (5%) membranes and (**b**) cross-linked HEC/SA^CA^, HEC/SA^PA^, and HEC/SA^CaCl2^ membranes.

Thermochemical properties of the membranes were investigated by TGA. The thermograms (TG) for the untreated and cross-linked HEC/SA and HEC/SA/fullerenol (5%) membranes, and cross-linked HEC/SA^CA^, HEC/SA^PA^, and HEC/SA^CaCl2^ membranes are presented in Figure 11.

The TG curves of untreated membranes in Figure 11a demonstrate three stages of weight loss. The first stage of weight loss takes place up to 220 °C and is associated with the absorbed water molecules [88]. The weight loss in this area is small and equal to 12% for the HEC/SA and HEC/SA-5 membranes. The second stage of weight loss occurs from 220 to 290 °C. This stage can be attributed to the thermal decomposition of carboxyl and hydroxyl groups of the polymers [89]. The weight loss was 49% for HEC/SA and 48% for HEC/SA-5 membranes at 290 °C. Meanwhile, the third stage over 290 °C is attributed to the backbone degradation of HEC and SA [51,89]. Weight loss at 560 °C was 64% for the HEC/SA and 62% for HEC/SA-5 membranes. The cross-linking of the membranes with calcium chloride (Figure 11a) resulted in a flatter TG curve. The weight loss at 220 °C (the first stage) was 15% for the HEC/SA^CaCl2^ and for HEC/SA-5^CaCl2^ membranes, which was slightly higher than that for the untreated membranes, due to the larger amount of absorbed water in the modified membrane after the cross-linking process (long-term immersing in aqueous solution of the cross-linking agent). The second step of the weight loss for the cross-linked HEC/SA^CaCl2^ and HEC/SA-5^CaCl2^ membranes was from 220 to 370 °C, which indicated the strong cross-linking of the polymer chains and greater membrane stability compared to the untreated membranes. Weight loss at 560 °C was 68% for the HEC/SA^CaCl2^ and 66% for HEC/SA-5^CaCl2^ membranes. It is worth noting that thermal decomposition of the HEC/SA-5^CaCl2^ membrane takes place with lower weight loss compared to the HEC/SA^CaCl2^membrane, which indicates better thermochemical properties and more rigid structure due to the modification with fullerenol.

Figure 11b demonstrates the TG curves for the membranes cross-linked with various agents. These membranes are characterized by initially a dehydration process and decomposition steps of cross-linker and/or polymer backbone, the extent of which depends mainly on the cross-linking degree and the cross-linking agent nature [76]. There are three stages of weight loss for the HEC/SA^CA^ and HEC/SA^CaCl2^ membranes and only two stages of weight loss for the HEC/SA^PA^ membrane. The least weight loss is exhibited by the membrane cross-linked with phosphoric acid–HEC/SA^PA^ membrane (only 50% at 560 °C). This indicated the maximum degree of cross-linking of the polymer chains, resulting in the lowest membrane performance (Figure 2). The membrane cross-linked with citric acid (HEC/SA^CA^) has the largest weight loss (70% at 560 °C), whereas the membrane cross-linked with calcium chloride (HEC/SA^CaCl2^) has maximum thermal stability. For the HEC/SA^CaCl2^ membrane, the temperature range of the second stage is 220–370 °C, while for the membranes cross-linked with phosphoric and citric acids is 180–220 °C and above 220 °C, respectively.

To interpret the transport properties of the developed dense membranes, it was necessary to study the swelling degree of the membranes in components of the separated mixture. The hydrophilic–hydrophobic properties of the membrane surface were investigated by the contact angle of water measurements, to evaluate the penetration of components through the membrane. The contact angle of water and swelling degree in water and azeotropic mixture (12/88 wt.% water/isopropanol) for the developed membranes are presented in Table 5. It was demonstrated that swelling degree in isopropanol for all membranes did not exceed 3%, data not presented.

The untreated membranes (HEC/SA and HEC/SA-5) collapsed in water, which also prevented the measurement of their contact angles of water. The cross-linkage of the HEC/SA blend was confirmed by swelling experiments. The cross-linking of these membranes with CaCl_2_, PA, and CA stabilized them in pure water and allowed applying them in pervaporation of isopropanol-water mixture in the wide concentration range (12–100 wt.% water) (Figure 2). The HEC/SA^CaCl2^ membrane had the highest swelling degree in water (207%) and the water-isopropanol azeotropic mixture (26%), and the lowest contact angle of water (35°) compared to other cross-linked unmodified membranes. This largely led to an increased performance of this membrane because of the accelerated diffusion of components through the membrane and a more hydrophilic surface, which facilitated the sorption of water molecules. The introduction of 5 wt.% fullerenol into the HEC/SA matrix (the untreated HEC/SA-5 membrane) led to a slight increase of swelling degree in the azeotropic mixture (23%) compared to the untreated HEC/SA membrane, which could be caused by a large number of polar -OH groups of fullerenol. However, for the cross-linked membranes by CaCl_2_ opposite trend was observed. The decreasing of swelling degree in water (154%) and the azeotropic mixture (18%) of the modified HEC/SA-5^CaCl2^ membrane compared to the HEC/SA^CaCl2^ membrane (207% and 26%, respectively) could be explained by the cross-linking of polymer chains both with calcium chloride and fullerenol. The effective cross-linking also contributed to the maintaining of the high selectivity of the modified membrane with significantly improved permeability in the pervaporation of isopropanol dehydration (Figure 2).

Thus, based on the results obtained, it can be concluded that the increase in permeation flux of the HEC/SA-5^CaCl2^ membrane compared to the HEC/SA^CaCl2^ membrane was related to several factors. These include the hydrophilization of the membrane surface (contact angle of water was insignificantly lower), more structured and evenly roughened membrane structure, increased membrane surface roughness, and decreased CaCl_2_ cross-linking effect by fullerenol.

### 3.3. Study of Transport and Structural Properties of the Cross-Linked Supported Membranes

Based on the pervaporation experiments of isopropanol dehydration for the dense membranes (described earlier in Section 3.1), it was shown that the membrane modified with 5 wt.% fullerenol and cross-linked with CaCl_2_ (HEC/SA-5^CaCl2^) had the best transport characteristics. For the promising application of this membrane in industry, the improvement of permeability is required. It can be achieved by the decrease of membrane thickness. Moreover, the stability in pervaporation separation of the isopropanol-water mixture with the excess of water of the supported membrane should be excellent. Thus, the membrane cross-linked with CaCl_2_ with a thin selective layer based on HEC/CA/fullerenol (5%) deposited on a porous PAN substrate (HEC/SA-5/PAN^CaCl2^) was developed and investigated. The unmodified cross-linked supported HEC/SA/PAN^CaCl2^ membrane was also developed for comparison with the fullerenol-containing membrane. In addition, in order to vary the properties of the HEC/SA-5/PAN^CaCl2^ membrane depending on the separation tasks, this membrane was subjected to surface modification with polyelectrolytes by layer-by-layer assembly.

#### 3.3.1. Study of the Cross-Linked Supported Membranes

**Figure 12:**
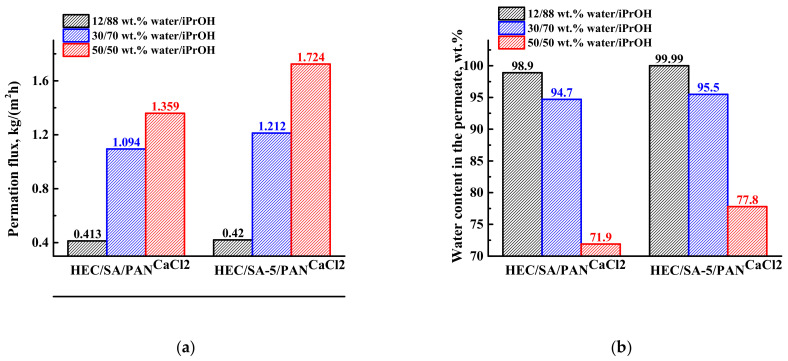

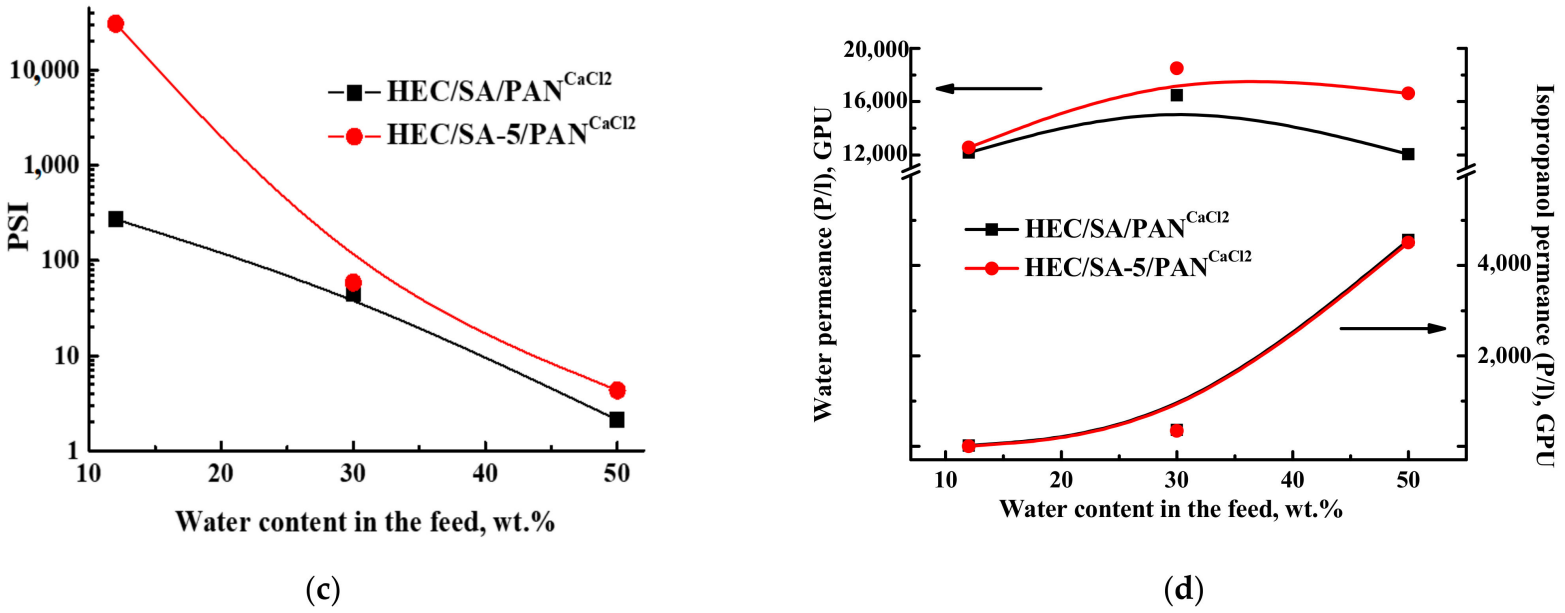
The dependence of (**a**) permeation flux, (**b**) water content in the permeate, (**c**) pervaporation separation index (PSI), and (**d**) component permeances on the water content in the feed for the supported cross-linked HEC/SA/PAN^CaCl2^ and HEC/SA-5/PAN^CaCl2^ membranes in pervaporation dehydration of isopropanol (12–50 wt.% water) at 22 °C.

The transport properties of the developed cross-linked supported HEC/SA^CaCl2^ and HEC/SA-5^CaCl2^ membranes were studied in pervaporation dehydration of isopropanol (12–50 wt.% water) at 22 °C. The results are presented in Figure 12. This concentration range is explored since pervaporation is often used in a hybrid process in combination with distillation, where the output mixture can contain high water content (usually up to 30 wt.%).

It was demonstrated that the development of the supported membranes led to the increase in permeation flux ~3 times compared with the dense membranes, but with the decrease of water content in the permeate. The reduction of water content in the permeate for the supported membranes may be related to active swelling of thin selective layer in feed components (preferably in water), resulting in mutual penetration of isopropanol and water through the supported membranes. The same effect (increase in permeability and slight decrease in selectivity compared to the dense membrane) was also observed for supported polyvinyl alcohol (PVA) membranes on polyacrylonitrile and aromatic polysulfone amide (UPM-20) substrates [68,69]. However, it is worth noting that the modification with fullerenol and cross-linking with calcium chloride (HEC/SA-5/PAN^CaCl2^ membrane) provide a higher permeation flux and higher water content in the permeate compared to the unmodified HEC/SA/PAN^CaCl2^ membrane (Figure 12a,b). This confirms that fullerenol acts as both a modifier and a cross-linking agent for the blend HEC/SA membrane. Moreover, to demonstrate the efficiency of pervaporation separation, the PSI values for the HEC/SA/PAN^CaCl2^ and HEC/SA-5/PAN^CaCl2^ membranes are presented in Figure 12c. PSI curve of the HEC/SA-5/PAN^CaCl2^ membrane is much higher compared to the HEC/SA/PAN^CaCl2^ membrane, indicating high efficiency of pervaporation separation with the application of this membrane. Component permeances (Figure 12d) also demonstrate that the modified HEC/SA-5/PAN^CaCl2^ membrane is more effective and selective, since its water permeance is higher and the isopropanol permeance is lower in comparison to the HEC/SA/PAN^CaCl2^ membrane. At low water concentration in the feed (12 wt.% water), the membranes are extremely selective (Figure 12b): both membranes have the water permeance of about 12,000 GPU, and the isopropanol permeance is below 16 GPU. The increase in water content in the feed led to the increase in both isopropanol and water permeances. This increase is caused by the effects of membrane surface roughness and membrane swelling in water, leading to membrane plasticization. With an increase in water content in the feed, the membranes swell more, causing a stronger spreading of polymer chains and the penetration of isopropanol molecules together with water resulting in the decrease of selectivity of the membrane. Thus, the plasticization effect increases the water permeance less, but has a much larger effect on isopropanol permeance [82].

**Figure 13:**
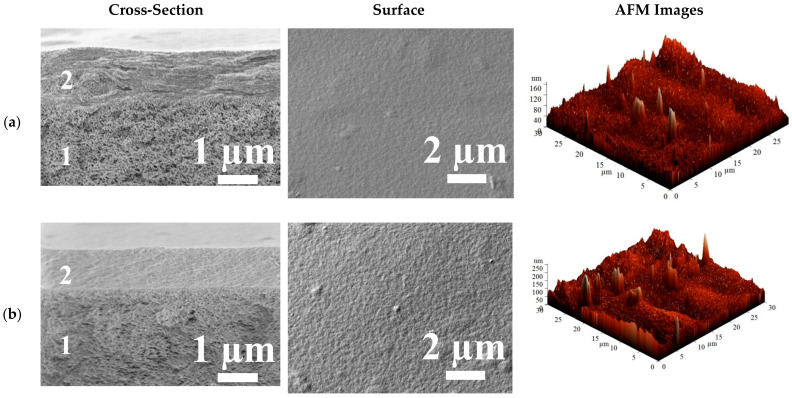
The cross-sectional and surface SEM micrographs, AFM images of the cross-linked (**a**) HEC/SA/PAN^CaCl2^ and (**b**) HEC/SA-5/PAN^CaCl2^ membranes.

The surface topography and the morphology of the cross-linked supported HEC/SA/PAN^CaCl2^ and HEC/SA-5/PAN^CaCl2^ membranes were also investigated using SEM and AFM. SEM cross-sectional and surface micrographs and AFM images (scan size of 30 × 30 μm) of these membranes are presented in Figure 13.

The cross-sectional SEM micrographs demonstrate the formation of uniform upper thin selective layers (2) with the thickness of ~1 ± 0.3 µm and their excellent adhesion to the porous PAN substrate (1) for the supported HEC/SA/PAN^CaCl2^ and HEC/SA-5/PAN^CaCl2^ membranes. Moreover, it should be noted that the modified HEC/SA-5/PAN^CaCl2^ membrane had more structured cross-sectional and rougher surface structure of the selective layer compared to the HEC/SA/PAN^CaCl2^ membrane. This effect was also observed for the dense HEC/SA^CaCl2^ and HEC/SA-5^CaCl2^ membranes (Figure 10). Based on AFM images for the supported membranes, the surface roughness parameters (R_a_ and R_q_) were calculated (Table 6).

It was demonstrated that the values of R_a_ and R_q_ parameters of the modified HEC/SA-5/PAN^CaCl2^ membrane were ~1.9 times higher compared with the unmodified HEC/SA/PAN^CaCl2^ membrane, which was in line with the SEM data for the membranes (Figure 13). The increased surface roughness of the HEC/SA-5/PAN^CaCl2^ membrane promotes the formation of a larger number of sorption centers, causing the improved permeation flux (Figure 12a). It is also worth noting that the HEC/SA/PAN^CaCl2^ and HEC/SA-5/PAN^CaCl2^ membranes have significantly less membrane surface roughness (Table 6), compared to the dense HEC/SA^CaCl2^ and HEC/SA-5^CaCl2^ membranes (Table 4). The deposited thin selective layers of supported membranes had a uniform and defect-free structure and good adhesion to PAN substrate according to SEM micrographs (Figure 13).

Contact angles of water for the supported HEC/SA/PAN^CaCl2^ and HEC/SA-5/PAN^CaCl2^ membranes were also evaluated (Table 6). It was demonstrated that the supported membranes had higher values of contact angles compared to the dense HEC/SA^CaCl2^ and HEC/SA-5^CaCl2^ membranes (Table 5). This observation may be attributed to the influence of the substrate nature on polymer crystallinity in the thin selective layer. It should be noted that the contact angle of water for the cross-linked by CaCl_2_ SA membrane was equal to 71° [77]. The decrease of contact angle of polymer blend can be related to the trapped HEC in SA that induces hydrophilic and smooth mixed matrix membrane surface [90]. Additionally, a slight decrease in the contact angle for the modified HEC/SA-5/PAN^CaCl2^ membrane compared to the HEC/SA/PAN^CaCl2^ membrane was observed. This was attributed to the introduction of fullerenol containing hydrophilic -OH groups into the membrane matrix that caused an increase of surface roughness and the formation of more sorption centers resulting to facilitated water membrane affinity [90]. The same effect was also observed for the dense membranes.

#### 3.3.2. Study of the Cross-Linked Supported Membranes with Surface Modification

**Figure 14:**
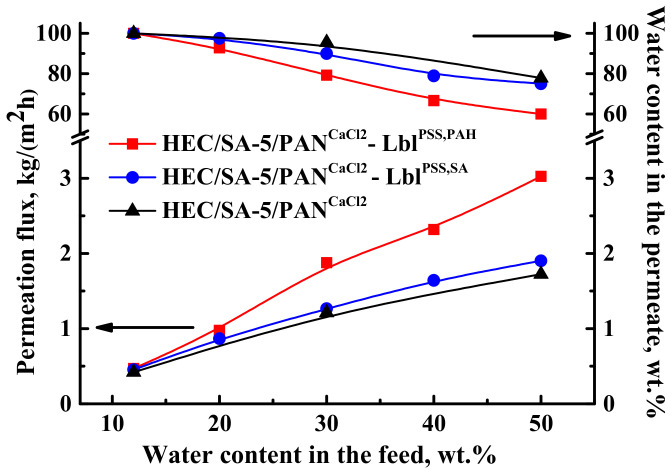
The dependence of permeation flux and water content in the permeate on the water content in the feed for the supported HEC/SA-5/PAN^CaCl2^, HEC/SA-5/PAN^CaCl2^—Lbl^PSS,PAH^, and HEC/SA-5/PAN^CaCl2^—Lbl^PSS,SA^ membranes in pervaporation dehydration of isopropanol (12–50 wt.% water) at 22 °C.

One of the simple and promising ways to improve the membrane properties is the deposition of polyelectrolytes (PEL) by Lbl assembly on the membrane surface [91,92,93]. This allows us to modulate transport properties of the membranes, depending on the separation tasks. The surface of the HEC/SA-5/PAN^CaCl2^ membrane was coated by 5 bilayers of various PEL pairs such as PSS/PAH and PSS/SA. This modification aimed to further increase the performance of the membranes in pervaporation dehydration, due to charge formation on the membrane surface, leading to an enhanced sorption of water maintaining high selectivity [68,69,74]. Transport properties of the HEC/SA-5/PAN^CaCl2^ membranes with surface modification (HEC/SA-5/PAN^CaCl2^—Lbl^PSS,PAH^, HEC/SA-5/PAN^CaCl2^—Lbl^PSS,SA^) were also tested in pervaporation dehydration of isopropanol till 50 wt.% water in the feed (Figure 14). The transport properties of the pristine supported HEC/SA-5/PAN^CaCl2^ membrane are also presented in Figure 14 for comparison.

It was shown that the deposition of 5 PSS/PAH bilayers on the surface of the HEC/SA-5/PAN^CaCl2^ membrane resulted in the highest permeation flux (0.4–3 kg/(m^2^h)) and the lowest water content in the permeate (till 60 wt.%) compared to other membranes. This effect may be explained by a higher level of surface roughness of membrane compared to the HEC/SA-5/PAN^CaCl2^—Lbl^PSS,SA^ membrane (confirmed by AFM data below). Increased roughness caused the formation of more hydrophilic mashes and more sorption centers, leading to increased membrane permeability, and the enhanced charge density of the membrane surface [71]. These factors induced reinforced penetration of the more polar component (water) resulting in the decrease of the selectivity [69,94]. At the same time, the HEC/SA-5/PAN^CaCl2^—Lbl^PSS,SA^ membrane demonstrated higher permeation flux of ~4–9% with the insignificant decreased water content in the permeate (up to 75 w.%) compared to the HEC/SA-5/PAN^CaCl2^ membrane. It should be also noted that for the supported HEC/SA-5/PAN^CaCl2^ and HEC/SA-5/PAN^CaCl2^—Lbl^PSS,SA^ membranes, water content in the permeate decreased significantly in the separation of the mixture containing over 30 wt.% water. For the HEC/SA-5/PAN^CaCl2^—Lbl^PSS,PAH^ membrane, it decreased significantly in the separation of the mixture containing over 20 wt.% water. Thus, we achieved the improvement of permeation flux of the supported HEC/SA-5/PAN^CaCl2^ membrane for pervaporation dehydration of isopropanol via surface modification with PEL. Depending on the separation purpose, the more efficient HEC/SA-5/PAN^CaCl2^—Lbl^PSS,PAH^ membrane (with the highest permeation flux) or the more selective HEC/SA-5/PAN^CaCl2^ (with good level of water content in the permeate) it can be selected for industrial dehydration.

**Figure 15:**
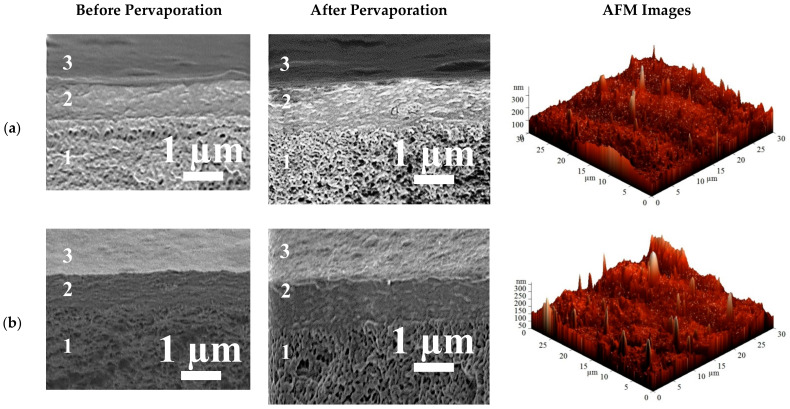
The cross-sectional SEM micrographs before and after pervaporation, AFM images of the (**a**) HEC/SA-5/PAN^CaCl2^—Lbl^PSS,PAH^ and (**b**) HEC/SA-5/PAN^CaCl2^—Lbl^PSS,SA^ membranes.

The surface topography of membranes modified by PEL was studied by AFM. The stability of the PEL layers on the membrane surface was studied by SEM and contact angle measurements. SEM cross-sectional micrographs before and after pervaporation and AFM images (scan size of 30 × 30 μm) for the HEC/SA-5/PAN^CaCl2^—Lbl^PSS,PAH^ and HEC/SA-5/PAN^CaCl2^—Lbl^PSS,SA^ membranes are presented in Figure 15.

The cross-sectional SEM micrographs of supported membranes with the surface modification before pervaporation demonstrate the presence of three areas: (1) the PAN substrate with the porous structure, (2) the thin nonporous selective layer based on SA-fullerenol (5%) composite (~1 µm thickness), and (3) the nanosized layer of PEL (PSS/PAH or PSS/SA) with the thickness of ~60 ± 10 nm. The cross-sectional SEM micrographs after pervaporation of the same membranes confirm that the polyelectrolyte layer is stable and does not wear off from the membrane surface after the experiments.

Based on the AFM images, the R_a_ and R_q_ parameters for the HEC/SA-5/PAN^CaCl2^—Lbl^PSS,PAH^ and HEC/SA-5/PAN^CaCl2^—Lbl^PSS,SA^ membranes were calculated (Table 7). The contact angles of water before and after pervaporation for the HEC/SA-5/PAN^CaCl2^—Lbl^PSS,PAH^ and HEC/SA-5/PAN^CaCl2^—Lbl^PSS,SA^ membranes are presented in Table 7.

It was demonstrated that the deposition of PEL layers on the surface of the HEC/SA-5/PAN^CaCl2^ membrane led to a slight increase in membrane roughness (compared to the data in Table 6). Additionally, the surface roughness of the HEC/SA-5/PAN^CaCl2^—Lbl^PSS,PAH^ and HEC/SA-5/PAN^CaCl2^—Lbl^PSS,SA^ membranes with different composition of PEL layer is comparative in values (Table 7). Minor changes in the contact angle values of the HEC/SA-5/PAN^CaCl2^—Lbl^PSS,PAH^ and HEC/SA-5/PAN^CaCl2^—Lbl^PSS,SA^ membranes before and after pervaporation also confirms the presence and stability of PEL layers on the membrane surface. Thus, the developed membranes with surface modification can be used for promising pervaporation dehydration applications.

### 3.4. Comparison of the Performance of the Developed Membranes with the Membranes Described in the Literature

Transport properties of three developed HEC/SA-5/PAN^CaCl2^, HEC/SA-5/PAN^CaCl2^—Lbl^PSS,PAH^, and HEC/SA-5/PAN^CaCl2^—Lbl^PSS,SA^ membranes were compared with the SA-based membranes described in the literature for the pervaporation dehydration of isopropanol. The studies using close conditions to the present study are summarized in Table 8.

It was demonstrated that the HEC/SA-5/PAN^CaCl2^ membrane developed in this study had the improved permeation flux with high selectivity in pervaporation separation of azeotropic water-isopropanol mixture (12/88 wt.%) compared to transport parameters of dense SA-based membranes in pervaporation dehydration of isopropanol with low water content (12.5 or 10 wt.% water). In pervaporation separation of water-isopropanol mixture (30/70 wt.%), this membrane also demonstrated improved permeation flux and the relatively high water content in the permeate (95.5 wt.%) compared to the SA-based membranes described in literature. Separately, it would be desirable to compare the results obtained in this article with our earlier published study [63] devoted to the development of supported membranes with selective layer from sodium alginate and its composite with fullerenol on a PAN substrate, cross-linked by CaCl_2_. In comparison, it can be seen that the addition of HEC to SA increases membrane permeability (under pervaporation separation of 30–50 wt.% water-isopropanol mixture the permeation fluxes were 1.1–1.4 kg/(m^2^h) and 0.95–1.10 kg/(m^2^h) for the HEC/SA/PAN^CaCl2^ and SA/PAN^CaCl2^ membranes, correspondingly). Moreover, under higher permeation flux, HEC-containing membranes had higher thickness (1 µm) as compared to SA supported membranes (0.6 µm). This result can be caused by increased swelling of the HEC-containing membrane in water in diluted solutions and higher surface parameters (Ra and Rq).

Additionally, transport properties of the HEC/SA-5/PAN^CaCl2^—Lbl^PSS,PAH^ and HEC/SA-5/PAN^CaCl2^—Lbl^PSS,SA^ membranes with surface modification were compared to the supported PVA-based membranes also modified by Lbl assembly with the same or similar polyelectrolytes for the pervaporation dehydration of isopropanol (Table 9).

The developed HEC/SA-5/PAN^CaCl2^—Lbl^PSS,PAH^ and HEC/SA-5/PAN^CaCl2^—Lbl^PSS,SA^ membranes are more productive and effective compared to supported PVA-based membranes with surface modification by Lbl assembly in pervaporation dehydration of isopropanol. However, this effectiveness gain is accompanied by a loss of selective properties (lower separation factor). It is also worth noting that the HEC/SA-5/PAN^CaCl2^—Lbl^PSS,PAH^ and HEC/SA-5/PAN^CaCl2^—Lbl^PSS,SA^ membranes are also characterized by sufficient water content in the permeate (79 and 90 wt.%, respectively), in separation of the isopropanol-water (70/30 wt.%) mixture.

Thus, in this work, high-performance and environmentally supported HEC/SA membranes have been developed for the efficient dehydration of isopropanol. These membranes are promising for industrial applications for the dehydration of other organic substances.

## 4. Conclusions

Novel highly efficient membranes based on polymer blend HEC/SA for pervaporation dehydration were developed using bulk and surface modifications. The effect of the HEC/SA ratio on the membrane transport properties in pervaporation dehydration of isopropanol (12 wt.% water) was investigated. It was shown that the HEC/SA (30/70) membrane had the optimal transport properties due the roughest and tortuous membrane structure and the highest surface roughness (demonstrated by SEM and AFM) caused by the optimal polymer distribution and uniform distribution of polar groups in the system (confirmed by FTIR and NMR spectroscopies). The modification of HEC/SA with fullerenol led to the increase in permeation flux by 17%, maintaining high selectivity to water (99.99 wt.% in the permeate), in pervaporation dehydration of isopropanol (12 wt.% water). This effect was related to fullerenol’s acting as a modifier increasing surface roughness (demonstrated by AFM) and functionalizing the surface (fullerenol particles as additional sorption centers on the membrane surface, SEM data). Besides, fullerenol acted as a cross-linking agent forming hydrogen bonds with the polymers (confirmed by FTIR). For the application of the dense membranes in pervaporation dehydration of isopropanol in a wide concentration range (12–100 wt.% water), chemical cross-linking with various agents (CaCl_2_, PA, and CA) was tested. Among dense untreated and cross-linked membranes, the membrane based on HEC/SA/fullerenol (5%) composite cross-linked with CaCl_2_ (HEC/SA-5^CaCl2^) demonstrated the highest permeation flux (increased by 30–50% compared to the HEC/SA^CaCl2^ membrane) and constant 99.99 wt.% water content in the permeate. Such high selectivity was associated with the effect of fullerenol: decreasing the cross-linking effect of CaCl_2_, increasing membrane surface hydrophilicity and roughness, and causing a structured and evenly roughened membrane.

To increase the performance of the higher performing modified dense cross-linked membrane for pervaporation dehydration of isopropanol, the supported membrane consisting of thin selective layer deposited on the PAN substrate was developed (HEC/SA-5/PAN^CaCl2^). It allowed increasing the permeation flux ~2 times compared to the cross-linked modified dense membrane, but with the decrease of selectivity. The cross-linked supported HEC/SA-5/PAN^CaCl2^ membrane had 0.42–1.72 kg/(m^2^h) permeation flux, and 77.8–99.99 wt.% water content in the permeate in pervaporation isopropanol dehydration (12–50 wt.% water). Surface modification of the HEC/SA-5/PAN^CaCl2^ membrane by Lbl assembly with the deposition of 5 bilayers of PSS/PAH and PSS/SA resulted in the increased membrane productivity in pervaporation dehydration of isopropanol: the permeation flux increased up to 0.47–3.0 and 0.46–1.9 kg/(m^2^h), respectively. However, the decrease in water content in permeate was observed compared to the cross-linked supported HEC/SA-5/PAN^CaCl2^ membrane. Thus, two alternative solutions may be offered for industrial dehydration, depending on the separation task: either the HEC/SA-5/PAN^CaCl2^—Lbl^PSS,PAH^ membrane with the highest permeation flux or the most selective HEC/SA-5/PAN^CaCl2^ membrane.

## Figures and Tables

**Table 1:** The properties of chemicals.

Substance	M_r_, g/mol	Solubility in Water, g/100mL	Melting Temperature, °C	Density, g/cm^3^
Citric acid	192.1	133.0	153	1.665
Phosphoric acid	98.0	548.0	42	1.685
Calcium chloride	111.1	74.5	772	2.15
Isopropanol	60.1	-	−90	0.7851

**Table 2:** Developed membranes based on hydroxyethyl cellulose/sodium alginate (HEC/SA) (30/70 wt.%) blend.

Membrane	Type	Thickness, μm	Content of C_60_(OH)_22–24,_ wt.%	Cross-Linking Method
HEC/SA	dense	30	0	-
HEC/SA-5	dense	30	5	-
HEC/SA^CA^	dense	30	0	3.5 wt.% citric acid
HEC/SA^PA^	dense	30	0	3.5 vol.% phosphoric acid
HEC/SA^CaCl2^	dense	30	0	1.25 wt.% calcium chloride
HEC/SA-5^CaCl2^	dense	30	5	1.25 wt.% calcium chloride
HEC/SA/PAN^CaCl2^	supported	1	0	1.25 wt.% calcium chloride
HEC/SA-5/PAN^CaCl2^	supported	1	5	1.25 wt.% calcium chloride

**Table 3:** Transport parameters of the HEC/SA and HEC/SA-5 membranes in pervaporation separation of the azeotropic water (12 wt.%)–isopropanol (88 wt.%) mixture at 22 °C.

Membrane	Permeation Flux, kg/(m^2^h)	Water Content in the Permeate, wt.%
HEC/SA	0.122	99.99
HEC/SA-5	0.143	99.99

**Table 4:** Surface parameters of the HEC/SA membranes.

Membranes	R_a_, nm	R_q_, nm
HEC/SA (10/90)	9.3	13.7
HEC/SA (30/70)	17.0	24.9
HEC/SA (50/50)	10.6	15.7
HEC/SA-5	32.9	46.1
HEC/SA^CA^	23.2	29.6
HEC/SA^PA^	18.5	23.9
HEC/SA^CaCl2^	50.4	63.0
HEC/SA-5^CaCl2^	65.2	81.8

**Table 5:** Swelling degree and contact angle of water for the HEC/SA membranes.

Membranes	Swelling Degree, %		Contact Angle of Water, °
	Water	Azeotropic Water-Isopropanol (12/88 wt.%) Mixture	
HEC/SA	-	20	-
HEC/SA-5	-	23	-
HEC/SA^CaCl2^	207	26	35 ± 2
HEC/SA^CA^	179	22	72 ± 3
HEC/SA^PA^	124	24	76 ± 4
HEC/SA-5^CaCl2^	154	18	30 ± 3

**Table 6:** Surface parameters of the supported HEC/SA/PAN^CaCl2^ and HEC/SA-5/PAN^CaCl2^ membranes.

Membranes	R_a_, nm	R_q_, nm	Contact Angle of Water, °
HEC/SA/PAN^CaCl2^	10.1	13.6	43 ± 3
HEC/SA-5/PAN^CaCl2^	18.7	25.4	41 ± 2

**Table 7:** Surface parameters and contact angle of water before and after pervaporation (PV) for the HEC/SA-5/PAN^CaCl2^—Lbl^PSS,PAH^ and HEC/SA-5/PAN^CaCl2^—Lbl^PSS,SA^ membranes.

Membranes	R_a_, nm	R_q_, nm	Contact Angle of Water, °	
			Before PV	After PV
HEC/SA-5/PAN^CaCl2^—Lbl^PSS,PAH^	23.9	30.5	66 ± 4	68 ± 5
HEC/SA-5/PAN^CaCl2^—Lbl^PSS,SA^	22.2	30.3	65 ± 3	66 ± 4

**Table 8:** The comparison of the transport properties of the SA-based membranes in pervaporation dehydration of isopropanol.

Membranes	Water Content in the Feed, wt.%	Temperature, °C	Permeation Flux, kg/(m^2^h)	Separation Factor (β)	Reference
HEC/SA-5/PAN^CaCl2^	12	22	0.420	73,326	This study
HEC/SA (10/90)-ZSM-5(40) (10%)^GA + UFS^ *	12.5	30	0.22	14,000	[6]
HEC/SA (10/90) ^GA + UFS^ *	12.5	30	0.09	14,000	
SA-chitosan-wrapped MWCNT (2%)	10	30	0.218	6419	[88]
SA-gelatin (10%)	10	30	0.085	4277	[95]
SA-phosphotungstic acid modified by ammonium carbonate (10%)	10	30	0.316	8991	[96]
SA-phosphomolybdic acid (10%)	10	30	0.282	9028	[97]
HEC/SA-5/PAN^CaCl2^	30	22	1.212	50	This study
SA-PVA (5%)	30	30	0.226	49.5	[98]
SA-karayagum (15%)	30	30	0.486	1613	[99]
SA/poly(acrylamide)-grafted guar gum (75/25)	30	30	0.164	153	[100]
SA–polystyrene sulfonic acid-co-maleic acid	30	30	~0.223	~1800	[101]
SA–heteropolyacids (10%)	30	30	~0.263	~1200	[102]
SA-aluminum-containing mesoporous silica (20%)	30	30	0.256	∞	[103]

* GA + UFS, glutaraldehyde + urea-formaldehyde-sulfuric acid.

**Table 9:** The comparison of the transport properties of the membranes with the Lbl assembly surface modification in pervaporation dehydration of isopropanol.

Membranes	Water Content in the Feed, wt.%	Tem-re, °C	Permeation Flux, kg/(m^2^h)	Separation Factor (β)	Reference
HEC/SA-5/PAN^CaCl2^—Lbl^PSS,PAH^	30	22	1.876	9	This study
HEC/SA-5/PAN^CaCl2^—Lbl^PSS,SA^	30	22	1.264	21	This study
PVA–PAH (4.7%)/PAN—Lbl^PSS,PAH^ (10 bilayers)	20	20	0.061	3996	[68]
PVA–PAH (4.7%)/UPM—Lbl^PSS,PAH^ (10 bilayers)	20	20	0.261	9	
PVA-fullerenol(5%)-PAH (4.7%)/UPM—Lbl^PSS,PAH^ (10 bilayers)	20	22	0.286	246	[69]
PVA-fullerenol (5%)-CS (20%)/UPM—Lbl^PSS,CS^ (5 bilayers)	20	22	0.340	87	[74]

## Data Availability

Data is contained within this article.
